# Analysis of R-loop forming regions identifies *RNU2-2* and *RNU5B-1* as neurodevelopmental disorder genes

**DOI:** 10.1038/s41588-025-02209-y

**Published:** 2025-05-29

**Authors:** Adam Jackson, Nishi Thaker, Alexander Blakes, Gillian Rice, Sam Griffiths-Jones, Meena Balasubramanian, Jennifer Campbell, Nora Shannon, Jungmin Choi, Juhyeon Hong, David Hunt, Anna de Burca, Soo Yeon Kim, Taekeun Kim, Seungbok Lee, Melody Redman, Rocio Rius, Cas Simons, Tiong Yang Tan, Jamie Ellingford, Raymond T. O’Keefe, Jong Hee Chae, Siddharth Banka

**Affiliations:** 1 Division of Evolution, Infection and Genomics, School of Biological Sciences, Faculty of Biology, Medicine and Health, University of Manchester, Manchester, UK; 2 Manchester Centre for Genomic Medicine, St Mary’s Hospital, Manchester University NHS Foundation Trust, Health Innovation Manchester, Manchester, UK; 3 Division of Clinical Medicine, School of Medicine and Population Health, University of Sheffield, Sheffield, UK; 4 Sheffield Clinical Genomics Service, Sheffield Children’s NHS Foundation Trust, Sheffield, UK; 5 Leeds Clinical Genomics Service, Leeds Teaching Hospitals NHS Trust, Leeds, UK; 6 Nottingham Regional Genetics Service, Nottingham City Hospital Campus, The Gables, Nottingham, UK; 7 Department of Biomedical Sciences, Korea University College of Medicine, Seoul, Republic of Korea; 8 Wessex Clinical Genetics Service, Princess Anne Hospital, University Hospital Southampton NHS Trust, Southampton, UK; 9 Department of Genomic Medicine, Seoul National University Hospital, Seoul, Republic of Korea; 10 Department of Pediatrics, Seoul National University College of Medicine, Seoul National University Children’s Hospital, Seoul, Republic of Korea; 11 Centre for Population Genomics, Garvan Institute of Medical Research and UNSW Sydney, Sydney, New South Wales Australia; 12 Centre for Population Genomics, Murdoch Children’s Research Institute, Melbourne, Victoria Australia; 13 Victorian Clinical Genetics Services, Melbourne, Victoria Australia; 14 Murdoch Children’s Research Institute, Melbourne, Victoria Australia; 15 Department of Paediatrics, University of Melbourne, Melbourne, Victoria Australia; 16 Genomics England, London, UK

**Keywords:** Clinical genetics, Neurodevelopmental disorders

## Abstract

R-loops are DNA–RNA hybrid structures that may promote mutagenesis. However, their contribution to human Mendelian disorders is unexplored. Here we show excess de novo variants in genomic regions that form R-loops (henceforth, ‘R-loop regions’) and demonstrate enrichment of R-loop region variants (RRVs) in ribozyme, snoRNA and snRNA genes, specifically in rare disease cohorts. Using this insight, we report neurodevelopmental disorders (NDDs) caused by rare variants in two major spliceosomal RNA encoding genes, *RNU2-2* and *RNU5B-1*. These, along with the recently described *RNU4-2*-related ReNU syndrome, provide a genetic explanation for a substantial proportion of individuals with NDDs.

## Abstract (web summary)

Genomic analyses focused on regions that form R-loops identify rare mutations in *RNU2-2* and *RNU5B-1* in individuals with neurodevelopmental disorders.

## Main

**Fig. 1:**
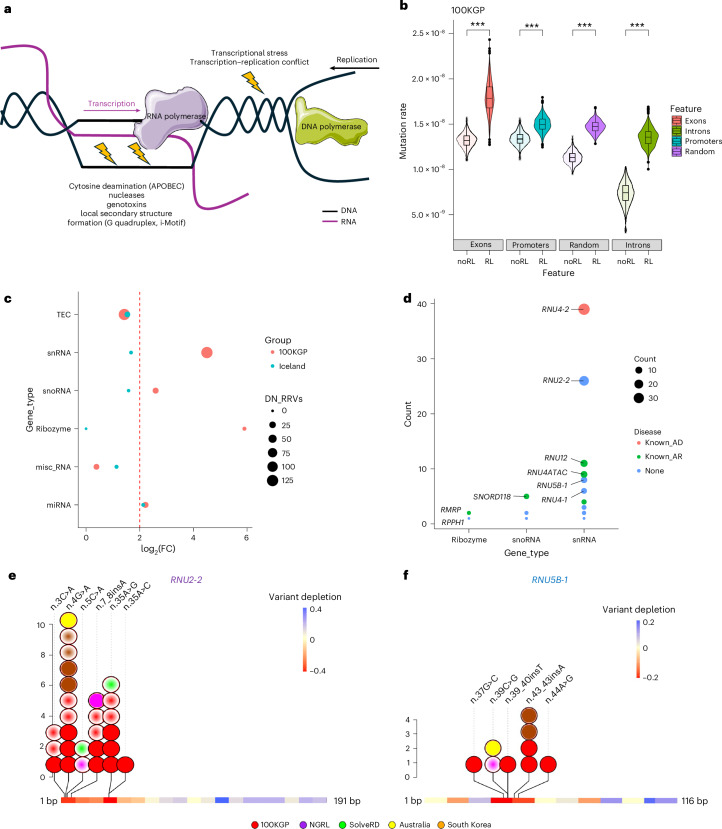
Analysis of variants in R-loop regions. **a**, Schematic of R-loops and the potential mutagenic processes that could result in increased mutagenesis in these regions. **b**, Violin plots showing mutation rate in 100KGP for genomic features that overlap (RL) or do not overlap (noRL) experimentally determined R-loop regions. We randomly selected 500 subset regions from each group for 1,000 iterations (***P < 2.22 × 10^−16^, two-sided Wilcoxon test). Violins extend from minimal to maximal data points. Box plots are centered on median with interquartile ranges as outer bounds, error bars as s.e.m and outliers as dots. **c**, DN RRV enrichment dot plot in GENCODE noncoding biotypes in 100KGP and Iceland control cohort. The red line marks the log_2_ fold enrichment threshold. **d**, Bubble plot of DN RRV enriched gene biotypes in 100KGP. **e**,**f**, Gene diagrams of *RNU2-2* (**e**) and *RNU5B-1* (**f**) with variant depletion as heatmap derived from gnomADv4. Variants are color coded by cohort of origin, with filled circles denoting DN variants and gradient-filled circles denoting unknown inheritance or parental transmission. Statistical data underlying the plots are provided as source data. Source data

**Extended Data Fig. 1:**
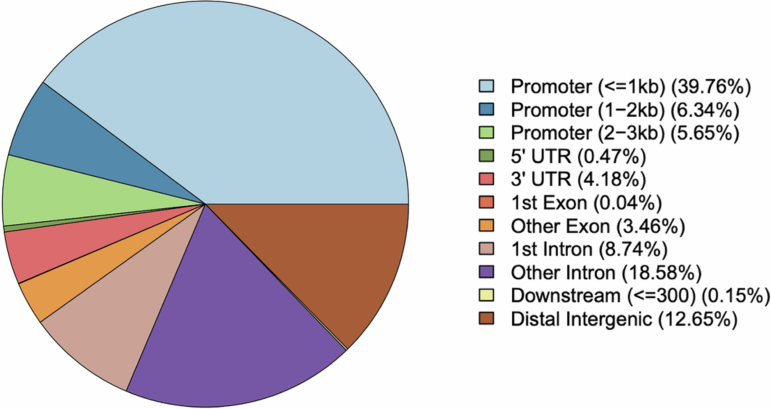
Genomic distribution of consensus experimentally determined R-loop regions. Pie chart produced by CHIPSeeker showing the distribution of R-loop consensus regions genome-wide.

**Extended Data Fig. 2:**
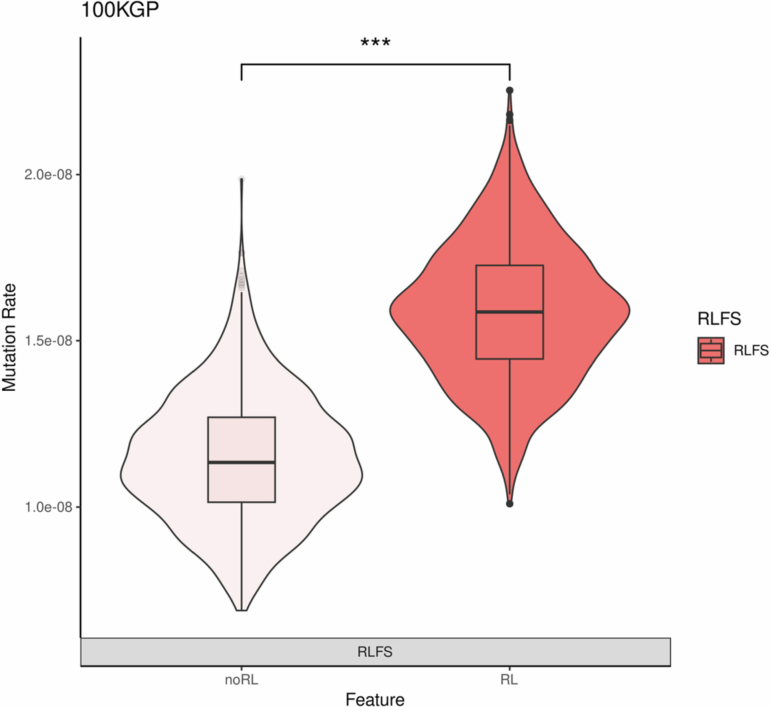
Rate of de novo variants in 100KGP rare disease cohort in predicted R-loop forming sequences. The ‘noRL’ group denotes regions that are predicted to form R-loops based on sequence context but lack experimental evidence. The ‘RL’ group denotes regions that are predicted to form R-loops based on sequence context and are supported by experimental evidence. ***P < 0.001 (two-sided Wilcoxon test, *n* = 1,000). Violins extend from minimal to maximal data points. Box plots are centered on median with interquartile ranges as outer bounds, error bars as s.e.m. and outliers as dots. Mutation rate represents de novo variants per bp per generation. Data underlying this plot are provided as source data. Source data

**Extended Data Fig. 3:**
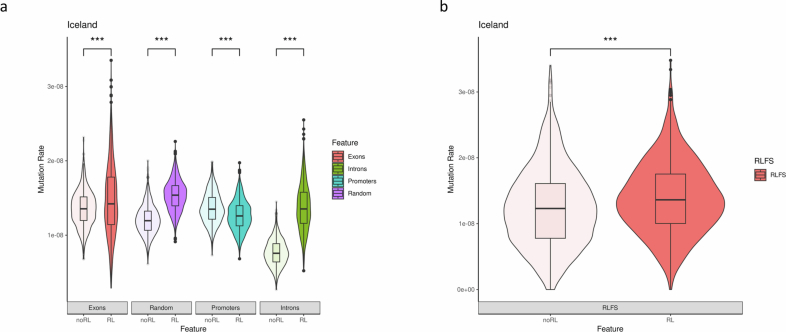
Rate of de novo variants in Icelandic healthy trio cohort in predicted R-loop forming sequences. **a**, Enrichment of de novo variants in RL regions in Icelandic cohort. ***P < 0.001 (two-sided Wilcoxon test, *n* = 1,000). Violins extend from minimal to maximal data points. Box plots are centered on median with interquartile ranges as outer bounds, error bars as s.e.m. and outliers as dots. Mutation rate represents de novo variants per bp per generation. **b**, Replication of enrichment for de novo variants in RLFS that overlap consensus R-loop regions. The ‘noRL’ group denotes regions that are predicted to form R-loops based on sequence context, but lack experimental evidence. The ‘RL’ group denotes regions that are predicted to form R-loops based on sequence context and are supported by experimental evidence. ***P < 0.001 (two-sided Wilcoxon test, *n* = 1,000). Violins extend from minimal to maximal data points. Box plots are centered on median with interquartile ranges as outer bounds, error bars as s.e.m. and outliers as dots. Mutation rate represents de novo variants per bp per generation. Data underlying these plots are provided as source data. Source data

R-loops form predominantly at sites of active transcription and may promote mutagenesis through exposure of single-stranded DNA to cytidine deaminases, nucleases, genotoxins or transcription–replication conflicts (Fig. 1a and Extended Data Fig. 1)^1–3^. Several methods for identifying R-loops exist^4^. By intersecting consensus R-loop regions (genomic footprint of 4.32%)^5^ with 975,406 variants in the 100,000 Genomes Project (100KGP)^6^ rare disease de novo (DN) dataset, we found 53,116 (5.4%, median = 4 per trio, range = 1–46) to be in R-loop regions. In regions that are well covered in gnomAD, we detected substantial excess of DN variants in R-loop versus non-R-loop regions of promoters, exons, introns and random genomic sequences (Fig. 1b). To test whether this observation could be due to R-loop regions having inherently higher sequence-context mutability, we ascertained all bioinformatically predicted R-loop-forming sequences and found the DN variant rate to be substantially higher in experimentally validated regions than in regions without experimental evidence, although the average mutability of the two groups was not significantly different (Extended Data Fig. 2 and Supplementary Table 1)^7^. All these findings, apart from excess DN RRVs in promoters, were validated in an independent 1,548 healthy Icelandic trios genome sequencing dataset (Extended Data Fig. 3)^8^. These results suggest that DN variants are more common in R-loop regions.

Next, we explored whether the distribution of DN RRVs is different in disease and control cohorts. We found significant genomic footprint-adjusted >2 log_2_ fold enrichment for DN RRVs exclusively in the rare disease cohort only for ribozyme, snoRNA and snRNA gene biotypes (Fig. 1c and Supplementary Table 2). These groups comprised of three distinct variants in two ribozyme genes, 15 variants in 12 snoRNA genes, and 86 variants in 18 snRNA genes (Fig. 1d and Supplementary Table 3), including five known recessive disease genes (Supplementary Table 4) and dominant ReNU syndrome (OMIM 620851) associated *RNU4-2* (*n* = 36)^9,10^. These results validated our approach to detect disease-causing variants.

**Extended Data Fig. 4:**
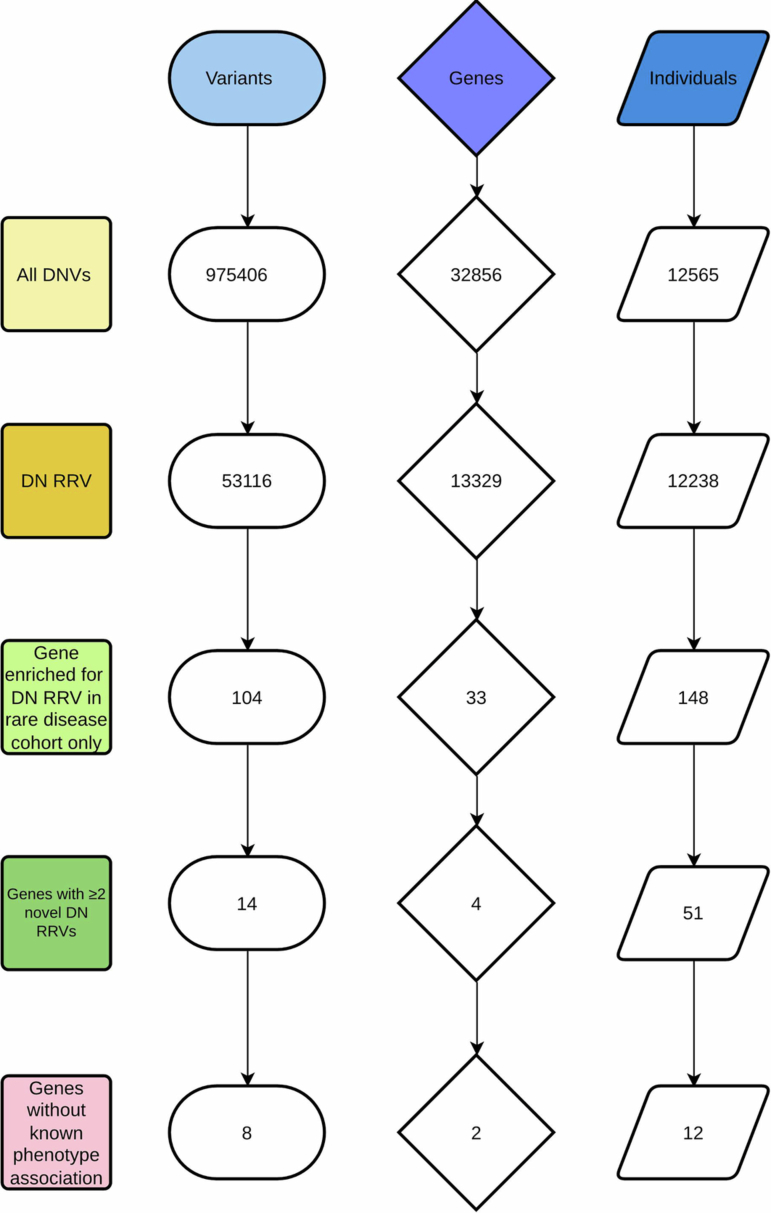
Filter logic to identify new disease genes in this study. Numbers of variants at each step are shown in lozenge shapes, number of genes in diamond shapes and individuals in parallelograms. For example, the last stage shows the identification of eight variants in two new dominant disorder genes (*RNU2-2* and *RNU5B-1*) in 12 individuals.

**Extended Data Fig. 5:**
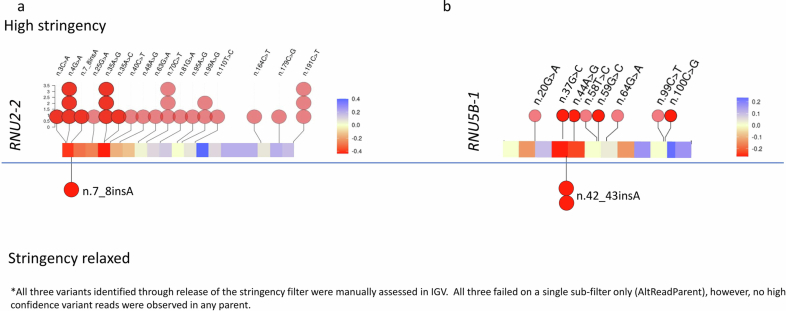
De novo variants in R-loop regions affecting genes of interest with absence from gnomADv4. **a**, All de novo variants in *RNU2-2* identified in 100KGP. **b**, All de novo variants in *RNU5B-1* identified in 100KGP. The bottom lollipops on each plot are variants that failed stringent de novo filtering. Lightly shaded circles indicate the presence of that variant in gnomADv4.

We next focused on genes without known disease associations and two or more new (absent from gnomADv4) DN RRVs in the rare disease cohort—*RNU2-2* (formerly *RNU2-2P*; five variants in nine individuals) and *RNU5B-1* (four variants in four individuals; Extended Data Fig. 4). The 100KGP DN variant dataset was generated with high stringency such that some real variants passing the base filter (based on read depth and zygosity in each sample) are excluded as they may fail on one of the stringency filters. For the two genes of interest, therefore, we released the stringency filter and identified three additional individuals with DN *RNU2-2* or *RNU5B-1* variants (Extended Data Fig. 5). These data indicated that *RNU2-2* and *RNU5B-1* could be new disease genes.

**Extended Data Fig. 6:**
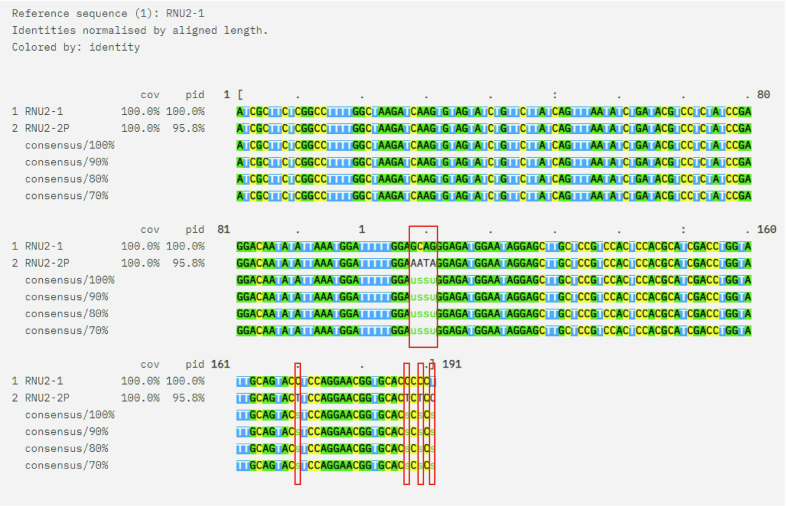
Sequence alignment of *RNU2-1* and *RNU2-2.* The eight nucleotide differences between *RNU2-1* and *RNU2-2* are highlighted by red boxes. Nucleotides are colored by identity.

We next investigated the distribution of DN RRVs detected in *RNU2-2* and *RNU5B-1* in the rare disease cohort and found that they occurred in regions constrained for variants in gnomADv4 (*n* = 76,215 individuals) and were absent from individuals coded as ‘unaffected’ (*n* = 32,030 participants) in 100KGP. This included five DN variants in a constrained region of *RNU2-2* (from 1 to 60 bp; ENST00000410396.1:n.3C>A, n.4G>A, n.7_8insA, n.35A>C and n.35A>G) in nine individuals (Fig. 1e). Notably, *RNU2-2* shares high sequence similarity with *RNU2-1* (Extended Data Fig. 6). *RNU2-1* resides in a 6.1-kb tandem repeat on chromosome 17 (ref. ^11^). This locus was not annotated in GRCh37 but is included in GRCh38 with thirteen repeat units. Importantly, eight nucleotides differ between *RNU2-2* and *RNU2-1*, allowing for unambiguous read alignment in GRCh38. For *RNU5B-1*, we found three DN variants in a constrained region (from 30 to 50 bp; ENST00000363286.1:n.37G>C, n.42_43insA and n.44A>G) in four individuals (Fig. 1f). Two individuals had new DN *RNU5B-1* variants outside of this constrained region, one of which (n.100C>G) had an alternative diagnosis that explained their phenotype. The other (n.59G>C) was an unsolved individual; however, this variant occurs in three other unaffected individuals in 100KGP. These data identify constrained regions in *RNU2-2* and *RNU5B-1* that may harbor disease-causing variants.

**Extended Data Fig. 7:**
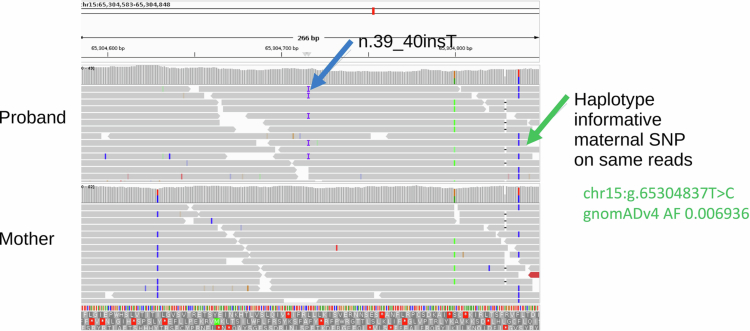
IGV screenshot of mother–daughter sequencing duo for individual #3. The *RNU5B-1* n.39_40insT variant is highlighted with a blue arrow. The informative maternal variant is highlighted by a green arrow. Note that in all the informative reads in the proband, the two variants are present in cis, showing that the proband’s *RNU5B-1* n.39_40insT variant is highly likely to have arisen on the maternal allele.

Next, we set out to expand the *RNU2-2* and *RNU5B-1* cohorts. Within the 100KGP database, we identified six other individuals with one of the abovementioned five *RNU2-2* heterozygous variants that were not DN or could not be proven to be DN (parental samples not available in four cases, and one instance each of maternal and paternal transmission). Expanding the search for other variants within the n.30–n.50 region of *RNU5B-1* identified one further individual in 100KGP with n.39_40insT, the DN origin of which was determined through phasing of maternal SNPs in this duo sequencing set up (Extended Data Fig. 7). Analysis of the Genomics England, National Genomics Research Library (NGRL; *n* = 32,203 genomes, 6,354 trios), SolveRD^12,13^ (*n* = 2,859 whole-genome sequencing (WGS)), the Centre for Population Genomics (Australia, *n* = 4,704 WGS, 864 trios) and South Korean Undiagnosed Diseases (*n* = 1,089 WGS probands) databases identified ten more individuals with rare *RNU2-2* variants and four with rare *RNU5B-1* variants in the constrained regions, bringing the total to 27 individuals for rare variants in *RNU2-2* (Fig. 1e) and nine for *RNU5B-1* (Fig. 1f).

**Fig. 2:**
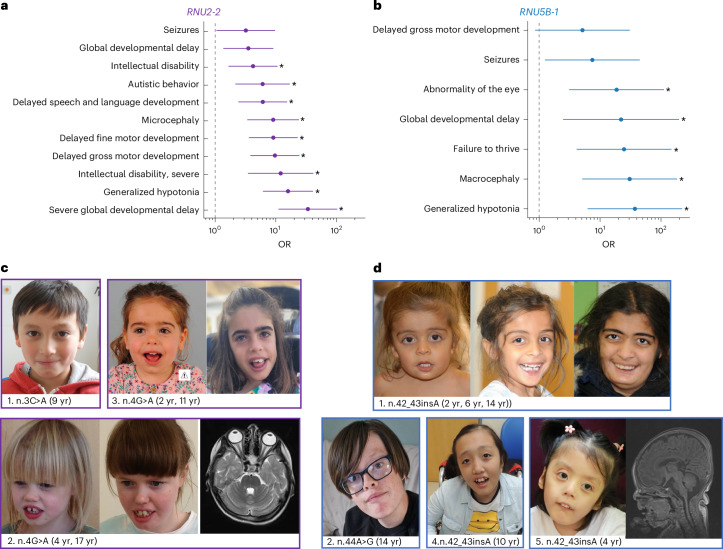
Clinical phenotype of individuals with *RNU2-2* and *RNU5B-1* variants. **a**,**b**, ORs for HPO terms in *RNU2-2* (**a**) and *RNU5B-1* (**b**) cases compared to all probands in the rare disease arm of 100KGP. Only HPO terms observed in at least three *RNU2-2* cases (*P < 0.0045, two-sided *Z* test of the log OR) or at least two *RNU5B-1* cases (*P < 0.0071, two-sided *Z* test of the log OR) are shown (exact *P* values are provided in Supplementary Table 10). **c**,**d**, Facial photographs and MRI images of affected individuals with *RNU2-2* (**a**) and *RNU5B-1* (**b**) variants. MRI brain of Individual 2 shows cerebral and cerebellar parenchymal volume loss. MRI brain of Individual 5 shows hypoplastic corpus callosum. Written informed consent for each individual was obtained from families for publication in this paper.

Next, we studied phenotypes of the *RNU2-2* and *RNU5B-1* cohorts. We found that all 21 probands with rare *RNU2-2* or *RNU5B-1* variants in 100KGP were previously genetically unsolved. Human Phenotype Ontology (HPO) terms including severe global developmental delay (odds ratio (OR) = 33.6, *P* < 10^−8^), generalized hypotonia (OR = 15.8, *P* = 1.2 × 10^−8^) and microcephaly (OR = 9.0, *P* = 1.1 × 10^−5^) were significantly enriched in individuals with *RNU2-2* variants in comparison with other rare disease probands (Fig. 2a). We were able to collect detailed clinical information from seven individuals with rare *RNU2-2* variants in our cohort, which revealed that the ‘*RNU2-2*-related disorder’ is characterized by global developmental delay, prominent speech impairment, epilepsy and, in some cases, presentation reminiscent of Pitt–Hopkins or Rett syndrome (Fig. 2b, Supplementary Tables 5 and 6 and Supplementary Note). Generalized hypotonia (OR = 37.3, *P* = 7.4 × 10^−5^), macrocephaly (OR = 30.6, *P* = 1.8 × 10^−4^), failure to thrive (OR = 24.7, *P* = 4.5 × 10^−4^), global developmental delay (OR = 22.1, *P* = 5.6 × 10^−3^) and abnormality of the eye (OR = 18.7, *P* = 1.4 × 10^−3^) were significantly enriched in individuals with *RNU5B-1* variants (Fig. 2c). We collected detailed clinical information from five individuals with rare *RNU5B-1* variants in our cohort, which revealed that the ‘*RNU5B-1*-related disorder’ is characterized by global developmental delay, relative macrocephaly, seizures and failure to thrive (Fig. 2d, Supplementary Tables 7 and 8 and Supplementary Note).

**Fig. 3:**
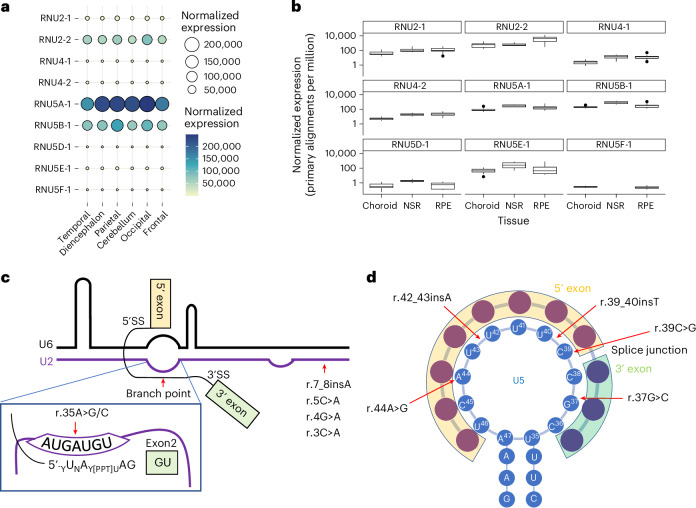
Characterization of *RNU2-2* and *RNU5B-1* variants. **a**, Balloon plot of small RNA-seq expression data with stringent multimapping protocol for RNU2, RNU4 and RNU5 paralogs in human developing brain derived from ENCODE. Normalized expression is in primary alignments per million. **b**, Box plots of small RNA-seq expression data with stringent multimapping protocol for RNU2, RNU4 and RNU6 paralogs in the human choroid (*n* = 13), neurosensory retina (NSR, *n* = 4) and retinal pigment epithelium (RPE, *n* = 16). Data are represented in box plots and the median value is central. **c**, Schematic representation of *RNU2-2* variants mapped to the U2–U6 structure in complex with the pre-mRNA branch point. **d**, Schematic representation of *RNU5B-1* variants mapped to the U5 structure in complex with the acceptor and donor sites of adjacent exons, amended from ref. ^20^.

As the predominant phenotypes of the two syndromes are neurodevelopmental, we asked whether the two genes are expressed in the developing human brain. As snRNAs are not polyadenylated, we used small RNA-seq data from ENCODE human developing brain and a related tissue, retina. We used a stringent bioinformatic protocol to remove multimapping reads as a confounder by including only primary alignments. Both datasets showed high expression of *RNU2-2* (higher than its multicopy paralog, *RNU2-1*) and *RNU5B-1* (Fig. 3a,b). Notably, *RNU2-2* was annotated as a pseudogene during the period of this study but encodes U2-2, a U2 small nuclear RNA that has previously been shown to be incorporated into the spliceosome, although its role remains putative^14,15^. Our small RNA-seq analysis shows that *RNU2-2* is highly expressed and not a pseudogene.

Next, we explored the effects of the six *RNU2-2* variants identified in the study. All variants are transversions or insertions affecting positions n.3, n.4, n.5 and n.7 located within the 5' end of U2 and predicted to disrupt interaction with the 3' end of U6 in the major spliceosome (Fig. 3c)^16^. The n.35A nucleotide is in the branch site recognition sequence (GUAGUA) that base-pairs with the conserved U of the pre-mRNA branch point (YNYURAY, where Y, pyrimidine; N, any nucleotide; and R, purine)^17^. A dominant-negative effect of variants at the yeast U2 36A (orthologous to human n.35A) position is supported by functional analysis in vitro that found the U2 36A>G variant does not support splicing^18^. Importantly, although none of the variants in our cohort are present in the general population, different heterozygous variants (n.3C>T and n.7_8insG) at the same positions have been reported in gnomADv4, suggesting that only specific variants in the constrained regions of *RNU2-2* may be pathogenic or that some may demonstrate reduced penetrance.

**Extended Data Fig. 8:**
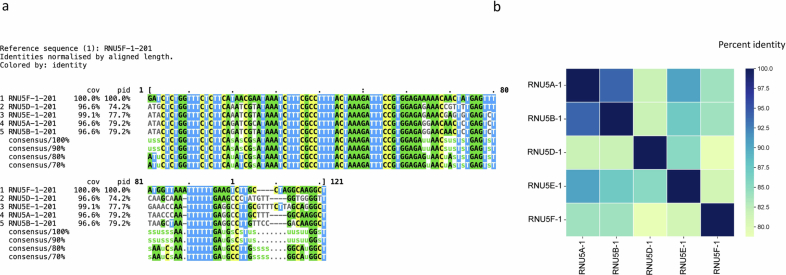
Multiple sequence alignment of U5 paralogs and their sequence identity. **a**, Multiple sequence alignment of human U5 paralogs normalized to *RNU5F-1*, which is the longest sequence. Nucleotides are colored by identity. **b**, Heatmap of percent identity for U5 paralogs showing that *RNU5A-1*, *RNU5B-1* and *RNU5E-1* share highest identity with each other.

We also studied the effects of the five *RNU5B-1* variants identified in the study. *RNU5B-1* encodes U5B-1, one of five U5 paralogs in the human genome (Extended Data Fig. 8). The *RNU5B-1* variant positions lie within the invariantly conserved loop I structure of U5, essential for holding and aligning the two exons for accurate joining during splicing (Fig. 3d)^19,20^. Notably, a different substitution in the constrained region, n.39C>T, is observed in three individuals in gnomADv4.

In summary, we show that DN variants are frequent in R-loop regions and that variants in snRNA components of the major spliceosome show enrichment in a disease cohort in comparison with a control cohort. We implicate rare variants in *RNU2-2* and *RNU5B-1* as causes for neurodevelopmental disorders (NDDs). *RNU2-2*-related disorder is characterized by global developmental delay, intellectual disability, microcephaly, autistic behavior and tendency for seizures. *RNU5B-1*-related disorder is characterized by global developmental delay, hypotonia, macrocephaly and failure to thrive. Along with variants in *RNU4-2*, variants in *RNU2-2* and *RNU5B-1* provide a genetic explanation for an exceptionally large proportion of individuals with NDDs caused by variants in nonprotein-coding genes and previously unsolved genetic NDDs (Supplementary Table 9). Our approach also demonstrates the utility of incorporating information about DNA secondary structures in variant analysis for identifying noncoding transcribed regions.

## Methods

The research presented here complies with all relevant ethical regulations and was performed under the approvals given by the South Manchester National Health Service (NHS) Research Ethics Committee (REC; 11/H1003/3/AM02), Cambridge South NHS REC (14/EE/1112–100KGP), University of Tübingen (ClinicalTrials.gov registration: NCT03491280–SolveRD), Seoul National University Hospital Institutional Review Board (2006-059-1131 and 2407-195-1559) and the Royal Children’s Hospital Human Research Ethics Committee (HREC/67401/RCHM-2020–Rare Diseases Now). Informed consent was obtained from all participants or their legal guardians.

### Genomic distribution of R-loop regions

To investigate the genomic distribution of R-loop regions, CHIPSeeker was used through the Galaxy platform to annotate R-loop regions based on their genomic context. GENCODE v.32 was used as the input gtf file.

### Cohort information

#### 100KGP

Participants were recruited to 100KGP with diverse rare disease phenotypes. Information about 100KGP is available at https://www.genomicsengland.co.uk/initiatives/100000-genomes-project and the preliminary report^6^. The rare disease cohort was derived from 73,527 genomes (release v19). The DN dataset was derived from 13,949 rare disease trios (active consent was available from 12,565 trios at the time of this study). The group of unaffected individuals in 100KGP was defined by keeping individuals with ‘unaffected’ as their status in the participant_summary table in LabKey.

#### NGRL

The NHS Genomic Medicine Service (https://re-docs.genomicsengland.co.uk/gms_release4/) data (30,047 rare disease genomes, including 15,889 from probands) in the NGRL was searched in the Genomics England Research Environment (GERE). Detailed information on indications for WGS in the NHS can be found at https://www.england.nhs.uk/publication/national-genomic-test-directories/. Of 15,889 probands, 6,321 (39.7%) were recruited for R27 (multiple congenital malformations and dysmorphic syndromes) or R29 (intellectual disability).

#### SolveRD

SolveRD sequencing data were accessed through the RD-CONNECT platform (https://rd-connect.eu/). SolveRD includes 523 genomes and 9,351 exomes from 9,645 individuals. Disease categories for the recruitment to SolveRD comprise rare neurological diseases (*n* = 2,271 families), (multiple) malformation syndromes, intellectual disability and other neurodevelopmental disorders (ITHACA and SpainUDP, *n* = 1,857), rare neuromuscular diseases (EURO-NMD, *n* = 1,517) and suspected hereditary gastric and bowel cancer (GENTURIS, *n* = 359). Only WGS datasets were included in this analysis, for which 334 correspond to affected individuals^21^. The proportion of each recruitment category with WGS is not available.

#### Australia

The Centre for Population Genomics hosts sequencing data for Australian individuals suspected of living with rare genetic conditions. Neurodevelopmental disorders are a major focus, although other key disease areas include epileptic encephalopathies, leukodystrophies, mitochondrial and renal conditions. As of this analysis, the dataset contains WGS data for 2,845 affected Individuals from 2,564 families (total Individuals with WGS data = 4,704). In 864 cases, full parental trio WGS was available^22^.

#### South Korea

A total of 3,976 participants were recruited from the Korea BioBank and a pediatric rare disease cohort comprising patients and their relatives from SNUH and its network hospitals. Among these, 1,150 individuals presented with NDDs, with or without epilepsy, that remained genetically undiagnosed despite prior microarray and exome sequencing analyses. To refine the cohort, kinship analysis was conducted to identify unrelated individuals, resulting in a final study cohort of 1,089 probands^23^.

### Identification and characterization of DN variants within R-loop regions in the 100KGP

The project was registered with the Genomics England research registry (RR1147) and received approval to access data from 100KGP (Genomics England, NGRL v.5.1: 10.6084/m9.figshare.4530893/v7).

Chromosomal coordinates, aligned to GRCh38, were downloaded from previously published consensus R-loop regions (RL regions) derived from 810 R-loop mapping datasets in humans^5^. These were converted to BED format and imported to GERE using the Airlock.

This BED file was intersected with a BED file generated from the DN variant dataset in GERE (main_programme_v18_2023-12-21) using the bedtools^24^ intersect function, with default parameters. To generate the de novo BED file, GRCh37 coordinates in the GERE were lifted over to GRCh38 using UCSC-liftOver, and then, the start position of each variant in the harmonized file was taken as the start position in the BED file and the end position was ‘start +1’. For initial analysis, only high-confidence DN variants in GERE flagged by a stringency criterion of ‘1’ were included. DN variants that failed the stringency filter were included for genes of interest and sequencing reads were manually inspected in IGV. Further information on the DN variant dataset in 100KGP can be found at https://re-docs.genomicsengland.co.uk/de_novo_data/. The DN dataset was generated with two quality filters—base and stringent. The base filter is applied on zygosity (heterozygous in the offspring and homozygous reference in the parents) and read depth (minimum 20× and maximum 98×) in all samples of the trio. If the variant fails any of these filters, it fails the base filter. Variants that pass base filter are then subject to the stringent filter, which include the following: altreadparent_filter (no more than one read supporting the alternate allele in either the mother or the father); abratio_filter (the AB ratio in the offspring is between 0.3 and 0.7); proximity_filter (DNV is not located within 20 bp of another DNV within the same trio); segmentalduplication_filter (no overlap with segmental duplications); simplerepeat_filter (no overlap with simple repeat regions; patch_filter (no overlap with patch regions); stringent_filter (**all base and stringent filters pass). Variants that failed the stringency filter in *RNU2-2* (a single instance of n.7_8insA) and *RNU5B-1* (two instances of n.42_43insA) failed due to the ‘altreadparent’ filter, although no good quality alternate reads could be identified in the sequencing data in IGV.

All data were exported through the Airlock for preparation of this paper.

### Analysis of DNMs occurring in the Icelandic control dataset versus 100KGP

The DNM file for the genome sequencing of 1,548 trios in the Icelandic study^8^ was downloaded and used to generate a BED file of genomic coordinates (GRCh38). This file was intersected with the GENCODE v32 GTF file to extract overlaps with genes and then the number of overlaps for each gene type in GENCODE was counted. We repeated this analysis for DNMs in 100KGP. We used the genomic footprint of each gene type to calculate the number of DNMs per bp per trio for each group. We then calculated the ratio of DNMs comparing 100KGP to Icelandic controls. For instance, if a biotype has a proportional footprint on 1% of the human genome in GRCh38, then 1% of DN variants would be expected to occur in this biotype, assuming a uniform distribution of DN variants. If this biotype actually contains 10% of all DN variants, then it is enriched tenfold compared to genomic footprint expectation. The expected number of DN RRVs was used as the probability for the binomial test, observed values as the success and the total number of DN RRVs as the trials. Adjustment for multiple testing was made by multiplying the *P* value by the number of GENCODE biotypes. Fold change was calculated as the number of observed DN RRVs divided by the expected number of RRVs for each biotype. Plots were generated in R using ggplot2 package. All source data are provided.

### Analysis of DNM enrichment in R-loop regions

A total of 50,000 random 1,000-bp genomic regions were generated from GRCh38 using bedtools random function. To generate regions with comparable selective pressure to R-loop regions, BED files were generated for all promoters and exons annotated by Ensembl regulatory build (GRCh38, 2016-11-11 and GENCODE v32, respectively). A BED file for GRCh38 introns was generated by subtracting the exon coordinates and overlapping with gene coordinates. Regions overlapping R-loop regions were identified using bedtools intersect and either removed with ‘-v’ option to generated ‘noRL’ BED files or selected to generate ‘RL’ BED files.

For each BED file, 500 random entries were selected (using shuf *.bed | head -n 500). These random sets of regions were separately intersected with 100KGP and Iceland DNM BED files. For each entry in the BED file, the number of overlapping DNMs was normalized to the entry genomic length, and then the sums of all regions were calculated and divided by the number of trios to give the total number of DNMs per bp per trio. The randomization was performed 1,000 times for each group—exons, promoters, introns and random. Results were plotted in R using ggplot2 as a grouped violin plot, and the ggsignif R package was used to calculate the Wilcoxon test *P* values between noRL and RL groups for each feature.

### R-loop forming sequence analysis

R-loop forming sequences (RLFSs) for GRCh38 were predicted by QmRLFS-finder^25^ and downloaded from the UCSC browser session provided in ref. ^5^. RLFSs were split into two groups—RLFS_RL, which overlap R-loop consensus regions and RLFS_noRL, which do not overlap consensus R-loop regions. This intersection was achieved using bedtools intersect. The subsampling procedure described above for genomic features was performed to calculate the mutation rate (DN variants per bp per generation). Statistical significance was calculated using the Wilcoxon test.

The mutability of RLFS_RL and RLFS_noRL was compared by extracting the Roulette annotations from the VCFs provided at http://genetics.bwh.harvard.edu/downloads/Vova/Roulette/. Where available, the adjusted mutation rate (AR) was extracted; otherwise, the raw score (MR) was used. The Roulette mutation rates for each possible SNV at each position within each RLFS were summed. The summed mutation rates were divided by the total number of all possible SNVs within each group to calculate the mean mutability per SNV for RLFS_RL and RLFS_noRL.

### Analysis of gnomADv4 coverage data

As coverage by WGS is reduced at repetitive regions, which may be annotated GENCODE biotypes or RLFS, we sought to identify regions of GRCh38 that were poorly covered in gnomADv4 to remove them from downstream analysis. We used the coverage data from gnomADv4 to select all nucleotides where fewer than 50% of individuals had 20× coverage. We then merged these into regions using bedtools merge with a flank of 20 bp, meaning that all poorly covered nucleotides within 20 bp of each other would be merged into a single region. We then removed these regions from downstream analysis using bedtools intersect -v.

### Analysis of variant depletion in gnomADv4

Due to the high mutability and strong selection pressures, the sliding window-based strategy^9^ was used to identify variant-depleted regions in the two genes. All PASS single-nucleotide substitutions in *RNU2-2* and *RNU5B-1* present in gnomADv4 were extracted using bcftools view -v snps *.vcf.gz | bcftools query -f ‘%CHROM %POS %REF %ALT\n’. A BED file with an entry representing each distinct variation in gnomADv4 was generated. Bedtools makewindows was used to generate 10-bp nonoverlapping windows for *RNU2-2* and *RNU5B-1*. Each gene window BED file was intersected with the gnomADv4 variant BED file and the number of overlaps for each window was counted. The number of possible substitutions was calculated for each window (nominally 30, for a 10-bp window, as each nucleotide has three possible substitutions). The number of observed substitutions in gnomADv4 was then divided by the number of possible substitutions. The resultant value was then normalized to the median of the window, meaning that strongly depleted regions have negative values, while variant-tolerant regions have positive values. Variant-depleted region was defined as one that had a normalized observed proportion of SNVs of less than −0.2.

### Analysis of gene snRNA expression in developing human brain using small RNA-seq

BAM files for small RNA-seq experiments in human brain, following treatment with tobacco acid phosphatase, were downloaded from ENCODE and aligned to GRCh38. Accession numbers for experiments used were ENCSR000AFR (diencephalon), ENCSR000AFY (parietal), ENCSR000AFX (occipital), ENCSR000AFS (frontal), ENCSR000AGD (temporal) and ENCSR000AFQ (cerebellum).

For each BAM file (two per tissue, representing two biological replicates), the total number of primary alignments for each gene was extracted using samtools view (‘samtools view -L gene.bed -c -F 260 $bam’). The total number of primary alignments in the BAM file (‘samtools view -c -F 260 $bam’) was then used to generate a normalized expression value by dividing the gene of interest primary alignment by all alignments, divided by 1 million (resulting in primary alignments per million). Data were plotted as balloon plots using ggplot2 in R.

### Analysis of gene snRNA expression in human retina

RNA was isolated from human donor eye tissue, which was collected and dissected as described previously^26^ from an ethically approved Research Tissue Bank (UK NHS Health Research Authority reference: 15/NW/0932). Total RNA was isolated from four neurosensory retina samples, 16 pelleted retinal pigment epithelium samples and 13 choroid samples that had been stored in RNAlater, using an Animal Tissue RNA Purification Kit (Norgen Biotek), as per the manufacturer’s instructions. Sequencing libraries were prepared using the NEBNext Multiplex Small RNA Library Prep Kit, as per the manufacturer’s protocols, with size selection performed using AMPure XP beads. Paired-end sequencing (2 × 75 bp) was performed on an Illumina HiSeq 4000.

NEBNext adapters were removed from sequencing reads using trimmomatic before alignment against the GRCh38 reference genome with bowtie. No mismatches between sequencing reads and the reference genome were allowed, and no restriction was set on multimapping reads. Sequence read counts were restricted to primary alignments using samtools v1.9, and therefore only counted once if they aligned to multiple *RNU2* or *RNU5* genes or pseudogenes. Calculations were drawn from read1 datasets and normalized for the total read count achieved for the sample.

### Multiple sequence alignment

Genomic fasta sequences for U2 and U5 paralogs were downloaded from Ensembl. Fasta sequences for *RNU2-1* (ENSG00000274585), *RNU2-2* (ENSG00000222328), *RNU5A-1* (ENSG00000199568), *RNU5B-1* (ENSG00000200156), *RNU5D-1* (ENSG00000200169), *RNU5E-1* (ENSG00000199347) and *RNU5F-1* (ENSG00000199377) were used. Alignments were performed using the Clustal Omega web tool and imported into MView for visualization. The percent identity matrix for U5 was imported into RStudio and the heatmap was plotted using the pheatmap package.

### Phenotype matching and analysis

Phenotypic information for individuals carrying candidate variants was extracted from the LabKey rare disease phenotype database (main_programme_v18_2023-12-21) using HPO terms. The analysis focused on identifying common phenotypes across individuals sharing these variants. For individuals with *RNU2-2* and *RNU5B-1* variants, the occurrence of each HPO term was counted, and ORs were calculated for term against all other individuals (*n* = 39,755) in the cohort. Significance thresholds were adjusted by Bonferroni correction.

### Reporting summary

Further information on research design is available in the Nature Portfolio Reporting Summary linked to this article.

## Online content

Any methods, additional references, Nature Portfolio reporting summaries, source data, extended data, supplementary information, acknowledgements, peer review information; details of author contributions and competing interests; and statements of data and code availability are available at 10.1038/s41588-025-02209-y.

## Supplementary information

Supplementary Information Supplementary Note.

Reporting Summary

Supplementary Tables Supplementary Tables 1–10.

## Source data

Source Data Fig. 1 Statistical source data for Fig. 1b.

Source Data Extended Data Fig. 2 Statistical source data.

Source Data Extended Data Fig. 3 Statistical source data.

## Data Availability

Genomic and phenotypic data are available for the 100KGP and individuals who have had WGS through the Genomic Medicine Service in the NGRL. Access to the NGRL may be granted following application via https://www.genomicsengland.co.uk/research/academic/join-research-network, which gives access to the secure GERE. Genomic data used pertain to participants in 100KGP in the Main Programme v.18 and the GMS data v.4. SolveRD data are accessible by application through the RD-CONNECT platform. Data presented in this paper were requested for the Airlock transfer on 26 September 2024. The paper was submitted for approval by the Genomics England Publication Committee on 27 September 2024 and was approved on 3 October 2024. Access to the Australian Centre for Population Genomics and South Korean Undiagnosed Diseases Program datasets can be requested through contact with the authors. The GRCh38 human genome reference assembly can be accessed at https://www.ncbi.nlm.nih.gov/datasets/genome/GCF_000001405.26/. The GENCODE v.32 comprehensive annotations were accessed within the GERE but can be downloaded from https://www.gencodegenes.org/human/release_32.html. The ENCODE data can be accessed at https://www.encodeproject.org/ and relevant accession codes are provided in Methods. The gnomADv4 genotype VCF files were accessed within the GERE but can also be downloaded from https://gnomad.broadinstitute.org/. Small RNA-seq datasets analyzed in this study are available at the NCBI Sequence Read Archive through accession PRJNA1256119. Source data are provided with this paper.

## Code availability

Software packages bedtools (v.2.31.0), bcftools (v.1.16) and samtools (v.1.9) were used in this work. Rv4 with RStudio, ggplot2 (v.3.5.2) and ggsignif (v.0.6.4) was used for plotting. Code generated for variant analysis within GERE is located within the secure GERE and available upon request.
