# Publisher Correction: Analysis of R-loop forming regions identifies *RNU2-2* and *RNU5B-1* as neurodevelopmental disorder genes

**DOI:** 10.1038/s41588-025-02274-3

**Published:** 2025-06-24

**Authors:** Adam Jackson, Nishi Thaker, Alexander Blakes, Gillian Rice, Sam Griffiths-Jones, Meena Balasubramanian, Jennifer Campbell, Nora Shannon, Jungmin Choi, Juhyeon Hong, David Hunt, Anna de Burca, Soo Yeon Kim, Taekeun Kim, Seungbok Lee, Melody Redman, Rocio Rius, Cas Simons, Tiong Yang Tan, Jamie Ellingford, Raymond T. O’Keefe, Jong Hee Chae, Siddharth Banka

**Affiliations:** 1 Division of Evolution, Infection and Genomics, School of Biological Sciences, Faculty of Biology, Medicine and Health, University of Manchester, Manchester, UK; 2 Manchester Centre for Genomic Medicine, St Mary’s Hospital, Manchester University NHS Foundation Trust, Health Innovation Manchester, Manchester, UK; 3 Division of Clinical Medicine, School of Medicine and Population Health, University of Sheffield, Sheffield, UK; 4 Sheffield Clinical Genomics Service, Sheffield Children’s NHS Foundation Trust, Sheffield, UK; 5 Leeds Clinical Genomics Service, Leeds Teaching Hospitals NHS Trust, Leeds, UK; 6 Nottingham Regional Genetics Service, Nottingham City Hospital Campus, The Gables, Nottingham, UK; 7 Department of Biomedical Sciences, Korea University College of Medicine, Seoul, Republic of Korea; 8 Wessex Clinical Genetics Service, Princess Anne Hospital, University Hospital Southampton NHS Trust, Southampton, UK; 9 Department of Genomic Medicine, Seoul National University Hospital, Seoul, Republic of Korea; 10 Department of Pediatrics, Seoul National University College of Medicine, Seoul National University Children’s Hospital, Seoul, Republic of Korea; 11 Centre for Population Genomics, Garvan Institute of Medical Research and UNSW Sydney, Sydney, New South Wales Australia; 12 Centre for Population Genomics, Murdoch Children’s Research Institute, Melbourne, Victoria Australia; 13 Victorian Clinical Genetics Services, Melbourne, Victoria Australia; 14 Murdoch Children’s Research Institute, Melbourne, Victoria Australia; 15 Department of Paediatrics, University of Melbourne, Melbourne, Victoria Australia; 16 Genomics England, London, UK

**Keywords:** Clinical genetics, Neurodevelopmental disorders

Correction to: *Nature Genetics* 10.1038/s41588-025-02209-y, published online 29 May 2025.

In the version of the article initially published online, there were typographical errors in the Fig. 1c *y*-axis label where “misc_RNA” appeared as “mise-RNA” and in the Fig. 2a *y*-axis label where “Microcephaly” appeared as “Macrocephaly.” The errors have been corrected in the HTML and PDF versions of the article.

